# (−)-Epicatechin-Enriched Extract from *Camellia sinensis* Improves Regulation of Muscle Mass and Function: Results from a Randomized Controlled Trial

**DOI:** 10.3390/antiox10071026

**Published:** 2021-06-25

**Authors:** Hyeyeong Seo, Seok-Hee Lee, Yooheon Park, Hee-Seok Lee, Jeong Sup Hong, Cho Young Lim, Dong Hyeon Kim, Sung-Soo Park, Hyung Joo Suh, Ki-Bae Hong

**Affiliations:** 1 Department of Integrated Biomedical and Life Science, Graduate School, Korea University, Seoul 02841, Korea; hishyung@korea.ac.kr; 2 Department of Food Science and Biotechnology, Dongguk University, Goyang 10326, Korea; seokhee@dongguk.edu (S.-H.L.); ypark@dongguk.edu (Y.P.); 3 Department of Food Science and Technology, Chung-Ang University, Anseong 17546, Korea; hslee0515@gmail.com; 4 Animal Center and Preclinical Evaluation Research Institute, Yonam College, Cheonan 31005, Korea; gangnaru@yonam.ac.kr; 5 R&D Center, BTC Corporation, Ansan 15588, Korea; cylim@btcbio.com (C.Y.L.); kdh@btcbio.com (D.H.K.); 6 Department of Food Science and Nutrition, Jeju National University, Jeju 63243, Korea; foodpark@jejunu.ac.kr; 7 Transdisciplinary Major in Learning Health Systems, Department of Healthcare Sciences, Graduate School, Korea University, Seoul 02841, Korea

**Keywords:** green tea, tannase, (−)-epicatechin, gallic acid, antioxidants, skeletal muscle

## Abstract

Loss of skeletal muscle mass and function with age represents an important source of frailty and functional decline in the elderly. Antioxidants from botanical extracts have been shown to enhance the development, mass, and strength of skeletal muscle by influencing age-related cellular and molecular processes. Tannase-treated green tea extract contains high levels of the antioxidants (−)-epicatechin (EC) and gallic acid that may have therapeutic benefits for age-related muscle decline. The aim of this study was to investigate the effect of tannase-treated green tea extract on various muscle-related parameters, without concomitant exercise, in a single-center, randomized, double-blind, placebo-controlled study. Administration of tannase-treated green tea extract (600 mg/day) for 12 weeks significantly increased isokinetic flexor muscle and handgrip strength in the treatment group compared with those in the placebo (control) group. In addition, the control group showed a significant decrease in arm muscle mass after 12 weeks, whereas no significant change was observed in the treatment group. Blood serum levels of follistatin, myostatin, high-sensitivity C-reactive protein (hs-CRP), interleukin (IL)-6, IL-8, insulin-like growth factor-1 (IGF-1), and cortisol were analyzed, and the decrease in myostatin resulting from the administration of tannase-treated green tea extract was found to be related to the change in muscle mass and strength. In summary, oral administration of tannase-treated green tea extract containing antioxidants without concomitant exercise can improve muscle mass and strength and may have therapeutic benefits in age-related muscle function decline.

## 1. Introduction

Over the past few decades, human life expectancy has shown an unprecedented increase owing to improved healthcare and living conditions. However, an extended lifespan has also resulted in an increased prevalence of age-related diseases, causing elderly healthcare to become an emerging social problem [1]. Among the various physiological changes associated with aging, age-related loss of skeletal muscle mass is the most common [2]. The decline in muscle function in the elderly is associated with an age-related reduction in muscle cells, imbalance between protein degradation and synthesis, inflammation-causing cytokines, cortisol, sex hormones, insulin resistance, and other factors related to lifestyle, such as nutritional intake and physical activity [3]. For instance, reduced physical activity with aging can lead to decreased muscle mass and functional ability, while vigorous physical activity can help to maintain overall health and muscle mass [4]. Identifying lifestyle interventions that can help maintain muscle mass and function can improve the health and quality of life of the elderly and reduce associated medical costs.

Oxidative stress resulting from increased reactive oxygen species (ROS) production and reduced scavenger protection has been implicated in age-associated muscular atrophy [5,6]. In addition, excessive ROS production causes DNA fragmentation, lipid peroxidation, and protein oxidation during aging, which can lead to cell apoptosis, further contributing to the loss of muscle mass and strength [7]. Therefore, inhibiting excessive ROS production is a potential therapeutic strategy to prevent muscle atrophy associated with long-term muscle inactivity in older individuals [8].

The understanding of the potential therapeutic benefits of food ingredients in disease prevention and management is an emerging area of research. Plant-derived antioxidants have been found to modulate the balance between ROS production and ROS scavenging through a variety of cellular mechanisms [9]. Bioactive compounds such as polyphenols, flavonoids, and phenolic acids have been found to attenuate ROS formation, lipid peroxidation, and inflammation in vitro and in vivo [10]. Polyphenols, for example, have been reported to mitigate stress-induced ROS production and regulate gene expression and the secretion of inflammatory cytokines [11]. Therefore, food and medicinal plants are important sources of exogenous antioxidants with potential therapeutic benefits [12].

Plant catechins have been shown to have multiple therapeutic benefits, including heart disease prevention, as well as anticancer and antibacterial effects [13]. They are also excellent antioxidants known for their ability to regulate muscle formation and counteract muscle atrophy [14,15]. In particular, green tea contains a large amount of polyphenol compounds such as (−)-epigallocatechin 3-gallate (EGCG), (−)-epigallocatechin (EGC), (−)-epicatechin 3-gallate (ECG), and (−)-epicatechin (EC), all of which have high antioxidant activity [16,17]. Although EGCG is the most well-known major component of green tea catechins, it has low bioavailability and is unstable in neutral or alkaline solutions [18]. As a result of this limitation, green tea EGCG needs to be converted into safer and more effective derivatives to exert its beneficial effects. EGCG can be converted into EC and gallic acid (GA) via treatment with the enzyme tannase. Compared to EGCG, the resulting GA shows increased bioavailability and antioxidant activity and has been found to inhibit body fat accumulation [19].

The use of tannase-treated green tea extract containing a high content of EC and GA has been reported to improve skeletal muscle recovery [20,21]. However, whether such beneficial effects occur in humans without added exercise is unknown. The purpose of this study was to evaluate the effect of tannase-treated green tea catechin extract on muscle function without concomitant exercise following twice daily ingestion of tannase-treated green tea extract for 12 weeks.

## 2. Materials and Methods

### 2.1. Materials

The tannase-treated green tea extract used in the randomized controlled trial was obtained from BTC Corporation (Ansan, Korea) by hydrolysis with the tannase enzyme, which was prepared as described in a previous study, with slight modifications [22]. Tannase-treated green tea extract was hydrolyzed at 40 ± 3 °C for 6–7 h by adding tannase enzyme (5 U/mL) to 12% green tea extract. The enzyme was then inactivated by heat treatment at 80 °C for at least 30 min. The HPLC analytical-grade standards of EGCG, EGC, ECG, EC, GA, and caffeine were purchased from Sigma-Aldrich (St. Louis, MO, USA) and were used to analyze the catechin content of the tannase-treated green tea extract.

### 2.2. Liquid Chromatography Analysis and Radical-Scavenging Assay

Analysis of the catechin content was performed using an HPLC system (Agilent Technologies 1260-series, Agilent, San Jose, CA, USA) and a UV detection system, as previously described [23]. The HPLC system for catechin, GA, and caffeine measurements consisted of a YMC-Triart C18 column (5 μm, 250 × 46 mm) and a UV/Vis detector. The injection volume of the sample was 10 μL, and the UV wavelength was 275 nm. The operating temperature was set at 35 °C, and the flow rate was 1.0 mL/min. The mobile phase was 0.1% phosphoric acid in water (mobile phase A) and 0.1% phosphoric acid in acetonitrile (mobile phase B). The gradient elution was 90% A + 10% B at 0–5 min, 87% A + 13% B at 5–10 min, 85% A + 15% B at 10–20 min, 70% A + 30% B at 20–25 min, 70% A + 30% B at 25–30 min, 90% A + 10% B at 30–31 min, and 90% A + 10% B at 31–35 min.

The antioxidant activity of the tannase-treated green tea extract was measured using 2,2′-azinobis (3-ethylbenzothiazoline-6-sulfonic acid) diammonium salt (ABTS, Sigma-Aldrich) and 2,2′-diphenylpicrylhydrazyl (DPPH, Sigma-Aldrich) assays. ABTS and DPPH radical-scavenging activities were measured using the method described by Brand-Williams and Re, respectively [24,25]. The radical-scavenging activity of the extract was expressed as IC_50_ (the concentration required to suppress the formation of radicals by 50%). Ferric reducing/antioxidant power (FRAP) and glutathione (GSH) were measured using methods described by Benzie and Jollow, respectively, with slight modifications [26,27].

### 2.3. Subjects and Study Design

Healthy Korean adults aged 60 years or older were recruited as subjects, and the study period was from June 2018 to April 2019. Subjects with the following conditions were excluded: (1) <0.8 m/s gait speed over 4 m; (2) grip strength ≤ 26 kg for men and ≤18 kg for women; (3) ≥30 kg/m^2^ body mass index (BMI); (4) clinically severe cardio-cerebrovascular, endocrine, neuropsychiatric, musculoskeletal, gastrointestinal, inflammatory, and/or hematologic/oncogenic diseases; (5) intake of drugs or health-functional food related to muscular function within one month before screening tests; (6) intake of antipsychotic agents within two months before screening tests; (7) alcoholism or drug abuse; (8) participation in other clinical trials in 2 months before screening tests; (9) aspartate transaminase or alanine transaminase levels three times higher than the upper limit, or serum creatinine > 2.0 mg/dL in the diagnostic test; (10) subjects determined by the investigator as unsuitable to participate in the study for other reasons. A total of 80 subjects who provided written consent were included in the study, of which 40 were assigned to the control group and 40 were assigned to the treatment group. The subjects were randomly assigned to receive either the test product (tannase-treated green tea extract 600 mg/day) or placebo (microcrystalline cellulose 600 mg/day). Each capsule contained tannase-treated green tea extract or other ingredients (cyclodextrin, magnesium stearate and caramel color), making up a total of 360 mg of the product (Supplementary Table S1). All subjects were recommended to take one capsule twice a day (morning and evening) for 12 weeks.

This was a single-center, randomized, double-blind, placebo-controlled study. After a screening test, subjects were registered on the basis of the inclusion criteria and assigned to either the treatment group or the placebo group. Subject blocks of certain sizes (e.g., 4, 6, and 8 subject blocks) were used for block randomization, and the codes for randomization were concealed from all subjects and investigators for the double-blind study until the end of the study. Out of a total of 80 subjects, 72 completed the test according to the protocol, four were withdrawn, and four were excluded due to violation of the protocol. Sixty-seven subjects (33 in the treatment group and 34 in the placebo group) were included in the analysis on the basis of initial isokinetic indicators and hormone levels. The study was approved by the Jeonbuk National University Hospital (No.: 2018-03-017) Institutional Review Board (30 April2018) in Korea and was conducted in accordance with the ethical standards of the Declaration of Helsinki (1964).

### 2.4. Analysis of Muscle-Related Factors

Physical examination was performed, vital signs (blood pressure and pulse rate) were assessed, and height (cm) and weight (kg) were measured to calculate the body mass index (kg/m^2^). Isokinetic muscular strength was measured using the Biodex System 3 pro (Biodex, Shirley, NY, USA). Peak torque of the extensor and flexor muscles of the knee joint was measured after practice and rest as the peak torque/body weight (TQ/BW) ratio at an angular velocity of 60°/s. Grip strength was measured using a Jamar dynamometer (Patterson Medical, Green Bay, WI, USA). Body fat and muscle mass were measured using dual-energy X-ray absorptiometry (DXA/Hologic Discovery, USA). Short physical performance battery (SPPB) was determined using a balance test, gait speed test, and chair stand test; the score of each test ranged from 0–4 [28]. Blood serum levels of follistatin, myostatin, interleukin (IL)-6, IL-8, insulin-like growth factor-1 (IGF-1), and cortisol were also examined using enzyme-linked immunosorbent assay [29].

### 2.5. Statistical Analyses

SPSS version 26 was used for all analyses. An independent *t*-test and a chi-square test were used to compare the general characteristics of the subjects in the placebo and treatment group. The independent *t*-test was used to evaluate the difference in isokinetic muscular strength between the treatment and control groups, while paired *t*-tests were used to evaluate the difference in this parameter at the baseline and at 12 weeks for each of these two groups. Grip strength, body muscle mass, biochemical variables, and hormones were analyzed in the same way. The level of significance was set at *p* < 0.05.

## 3. Results

### 3.1. Catechin Content and Antioxidant Activity of Tannase-Treated Green Tea Extract

**Figure 1:**
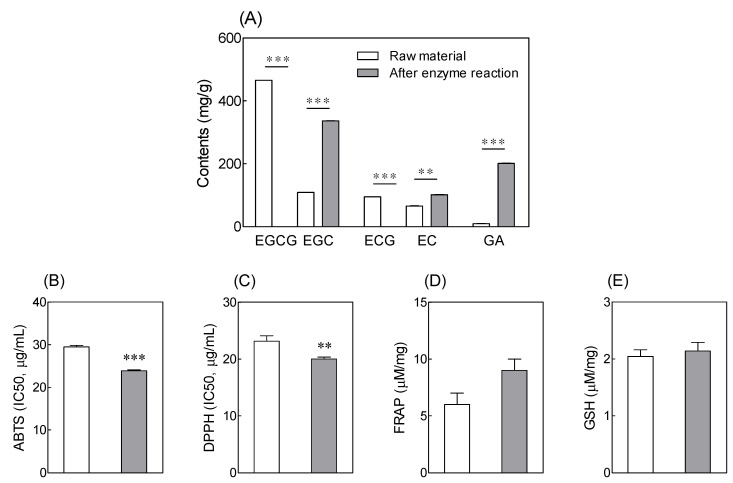
Effects of tannase treatment on (**A**) catechin contents, (**B**) ABTS, (**C**) DPPH, (**D**) FRAP, and (**E**) GSH contents in green tea extract. Values are represented as means ± standard deviation (SD). Different symbols indicate significant differences at *p* < 0.01 (**) and *p* < 0.001 (***) according to an independent-samples *t*-test. EGCG: (−)-epigallocatechin 3-gallate; EGC: (−)-epigallocatechin; ECG: (−)-epicatechin 3-gallate; EC: (−)-epicatechin; GA: gallic acid; ABTS: 2,20-azinobis (3-ethylbenzothiazoline-6-sulfonic acid) diammonium salt; DPPH: 2,2′-diphenylpicrylhydrazyl; FRAP: ferric reducing/antioxidant power; GSH: glutathione.

Differences in the total catechin content between the tannase-treated green tea extract and the untreated green tea extract were investigated (Figure 1A). The content of EC increased from 65.77 ± 0.90 to 101.35 ± 0.90 mg/g after tannase treatment, whereas that of GA increased from 9.93 ± 0.09 to 201.36 ± 0.77 mg/g. The antioxidant activities of both treated and untreated green tea extracts were measured using ABTS and DPPH radical-scavenging assays, FRAP, and GSH assays (Figure 1). ABTS and DPPH radical-scavenging abilities of tannase-treated green tea extract samples were 23.88 ± 0.17 and 20.03 ± 0.30 μg/mL, respectively. There was no significant difference between the treated and untreated green tea extracts for the FRAP and GSH assay results; however, ABTS and DPPH values confirmed that tannase treatment resulted in a significant increase in antioxidant activity of the extract.

### 3.2. Effects of Tannase-Treated Green Tea Extract on Isokinetic Muscular Strength

Out of a total of 80 subjects, eight were excluded from the analysis due to withdrawal or violation of the protocol, and five were excluded from the analysis on the basis of their initial muscle isokinetic index and hormonal levels. Thus, a total of 67 subjects (33 in the treatment group and 34 in the placebo group) were included in the analysis to investigate the differences between the control and treatment groups as a function of the protocol and initial isokinetic indicators and hormone levels. The mean age of the participants was 64.1 years, and 85.1% (*n* = 57) of them were women. Demographic data are summarized in Table 1. There was no significant difference in the baseline characteristics between the control and treatment groups. Thus, these groups were used to examine the effects of tannase-treated green tea extracts.

Isokinetic muscle strength tests were conducted at the baseline (before administration) and after 12 weeks of administration, and the results are summarized in Table 2. The peak torque (maximum muscle strength) of the flexor muscles of the right leg increased significantly after 12 weeks compared to that at the baseline in the treatment group that was administered the tannase-treated green tea extract (*p* = 0.027). In addition, there was a statistically significant difference compared to the control group (*p* = 0.013). The peak TQ/BW values for the flexor muscles of the right leg in the treatment group increased significantly after 12 weeks of administration compared to that at the baseline (*p* = 0.038), and there was a statistically significant difference between the treatment and control groups (*p* = 0.019).

### 3.3. Effects of Tannase-Treated Green Tea Extract on Grip Strength

The right-hand grip strength in the control group was significantly lower after 12 weeks compared to that at the baseline (Table 3, *p* = 0.004). In addition, the right-hand grip strength of the control group was significantly lower than that of the treatment group (*p* = 0.037). The left-hand grip strength tended to decrease after 12 weeks in the control group (*p* = 0.112); however, there was no significant difference when compared to the treatment group (*p* > 0.05).

### 3.4. Effects of Tannase-Treated Green Tea Extract on Body Muscle Mass

The differences in body muscle mass between those at the baseline and those after 12 weeks were measured using DXA analysis, and the results are summarized in Table 4. The muscle mass of the left and right arms in the control group decreased significantly after 12 weeks compared to that at the baseline (left arm: *p* = 0.004; right arm: *p* = 0.009). However, there was no significant difference between the control and treatment groups (*p* > 0.05). Trunk muscle mass significantly increased after 12 weeks in both the treatment and control groups (control group: *p* = 0.001; treatment group: *p* = 0.009); however, no significant difference was observed between the two groups (*p* > 0.05).

### 3.5. Effects of Tannase-Treated Green Tea Extract on Levels of Follistatin and Myostatin

The effect of tannase-treated green tea extract administration on the levels of follistatin, myostatin hs-CRP, IL-6, IL-8, IGF-1, and cortisol was confirmed through blood serum analysis. There was no significant change in the levels of follistatin, hs-CRP, IL-6, IL-8, IGF-1, and cortisol before and after 12 weeks in both the control and the treatment groups, and no significant difference was found between the groups (Table 5 and Supplementary Table S2). Myostatin levels significantly decreased after 12 weeks in the treatment group (Table 5, *p* = 0.007), and a statistically significant difference was observed between the groups (*p* = 0.045).

### 3.6. Safety Parameters and Adverse Events of Administration of Tannase-Treated Green Tea Extract

A total of 80 subjects provided written consent and were included in the safety analysis. Blood and urinary tests were conducted, and adverse events (AEs) were monitored to evaluate safety. Blood and urine parameters were recorded during the experimental periods, and none of the groups showed any significant differences (Supplementary Table S3). White blood cell count decreased in both groups after 12 weeks, but the results were within the normal range, and no statistically significant difference was observed between the groups. Forty-six AEs were reported in 34 subjects during the study. However, there was no statistically significant difference in the number of subjects with AEs between the groups (*p* > 0.05). No causal relationship between tannase-treated green tea extract administration and AE could be identified.

## 4. Discussion

Age-related loss of skeletal muscle function and mass (sarcopenia) adversely affects the quality of life in older individuals and is associated with several chronic illnesses. Decreased endogenous antioxidant efficiency, increased cytokine levels, DNA damage, and altered protein synthesis have all been implicated in the loss of muscle mass and strength with aging [30,31]. Dietary administration of plant-derived exogenous antioxidants has been found to mitigate age-related loss of muscle mass and function by scavenging ROS in the skeletal muscle [32]. Therefore, increased dietary intake of antioxidants may have therapeutic benefits in preventing and treating sarcopenia.

Green tea has well-known health benefits that have been attributed to its high content of antioxidant polyphenol catechins [33,34]. Hence, various methods for producing tea extracts with high levels of antioxidant activity and extractability have been attempted [35,36,37]. However, low bioavailability of green tea catechins limits the potential therapeutic benefits of green tea [18]. To improve its bioavailability and antioxidant activity, green tea EGCG needs to be converted to EC and gallic acid.

In this work, green tea extract was treated with the enzyme tannase. HPLC analysis and radical-scavenging assays were used for comparing catechin content and antioxidant activity of treated and untreated green tea extracts. Tannase-treated green tea extract displayed increased content of the catechins EC and GA. Additionally, the ABTS and DPPH assays revealed a significant increase in the antioxidant activity of the extract following tannase treatment. The enzyme-based extraction process is known to result in changes in the active compound content and antioxidant activity of plant materials [38,39].

We then investigated the effects of tannase-treated green tea extract containing antioxidants without concomitant exercise on isokinetic flexor muscle and handgrip strength. Administration of tannase-treated green tea extract for 12 weeks resulted in a significant improvement in muscle function (isokinetic flexor muscle and handgrip strength) in the treatment group compared with those in the placebo (control) group. Among the catechins present in green tea extracts, EC has been previously reported to mitigate muscle disease and muscle loss [40,41]. Continuous administration of EC in mice and humans has been reported to regulate fatigue resistance, oxidative capacity, angiogenesis, mitochondrial signaling, skeletal muscle growth, and differentiation [41,42,43]. Results from animal studies and clinical trials have also shown beneficial effects of antioxidant-rich catechins on recovery of physical performance, skeletal muscle aerobic capacity, differentiation of myogenic stem cells, biogenesis of mitochondria, and synthesis and degradation of proteins [15,44,45]. In addition, the combination of exercise and tea catechin supplementation improved muscle mass and physical functions such as walking ability in sarcopenic elderly women [14]. Attenuation of muscle damage by catechins has been associated with their ability to suppress oxidative stress and muscle inflammation [44,46].

Changes in body muscle mass and hormone levels were also assessed at two time points in both treatment and placebo groups and revealed a significant decrease in myostatin levels from the baseline after 12 weeks in the treatment group, but not in the placebo group. Myostatin is a negative regulator of muscle growth [47]. Increased levels of myostatin have been implicated in muscle wasting, and myostatin has been identified as a potential therapeutic target for sarcopenia [48,49,50,51]. The results obtained in this study suggest that green tea extract improves muscle mass and strength potentially by influencing myostatin levels. These observations are consistent with previous results from our group showing that EC administration resulted in a significant increase in the expression of muscle differentiation genes, such as myoblast determination protein (MyoD) and myogenin genes in stressed C2C12 muscle cells [20]. The levels of factors related to muscle degradation or loss, such as forkhead box protein O3 (FOXO3), myostatin, muscle RING finger protein-1 (MuRF-1), and atrogin-1, were also significantly reduced by treatment with green tea extract with a high EC content in aged mice [21]. Additionally, treatment of antioxidant-rich plant extracts resulted in increased levels of the muscle growth and production factors p70 S6 kinase (pS6K) levels and mammalian target of rapamycin (mTOR), along with the levels of follistatin, which inhibits myostatin [41,52,53].

The present study showed that administration of tannase-treated green tea extract for 12 weeks without added exercise significantly increased muscle and grip strength and muscle mass in individuals aged 60 years or older. Improved muscle function following administration of tannase-treated green tea extract was associated with a reduction in the levels of myostatin. To the best of our knowledge, this is the first evidence showing that antioxidant-rich tannase-treated green tea extracts improve muscle mass and function, even without concomitant exercise.

## 5. Conclusions

Enzymatic treatment increased the antioxidant capacity and the catechin content of green tea extract. A randomized controlled trial showed that 12 weeks of administration of tannase-treated green tea extract without any added exercise resulted in an increase in lower extremity muscle strength, suppression of grip strength reduction, and regulation of blood myostatin, compared to the placebo. These results show that tannase-treated green tea extract alone can lead to changes in muscle strength-related indicators without the need for concomitant exercise. These results suggest potential therapeutic benefits of tannase-treated green tea extract consumption for age-related muscle loss.

## Figures and Tables

**Table 1:** General characteristics of the subjects.

	Control Group (*n* = 34)	Treatment Group (*n* = 33)	Total (*n* = 67)	*p*-Value
Age (years)	63.5 ± 3.7	64.8 ± 3.7	64.1 ± 3.7	0.177
Sex				
Male (*n*, %)	5 (14.7)	5 (15.2)	10 (14.9)	0.959
Female (*n*, %)	29 (85.3)	28 (84.8)	57 (85.1)	
Height (cm)	157.0 ± 5.2	157.7 ± 6.4	157.3 ± 5.8	0.639
Weight (kg)	58.2 ± 9.3	61.2 ± 8.0	59.7 ± 8.8	0.158
BMI (kg/m^2^)	23.5 ± 2.9	24.6 ± 2.5	24.0 ± 2.8	0.121
SBP (mmHg)	125.9 ± 12.2	124.5 ± 14.0	120.9 ± 13.5	0.664
DBP (mmHg)	75.5 ± 7.8	77.2 ± 9.7	74.3 ± 10.3	0.665
Pulse (times/min)	73.5 ± 9.3	76.2 ± 9.7	72.7 ± 10.1	0.263
Alcohol (*n*, %)	8 (23.5%)	5 (15.2%)	13 (19.4%)	0.493
Smoking (*n*, %)	0 (0.0%)	1 (3.0%)	1 (1.5%)	0.306

Values are presented as the mean ± standard deviation. *p*-Values were determined by an independent *t*-test or chi-square test.

**Table 2:** Effects of tannase-treated green tea extract on isokinetic muscular strength.

			Control Group (*n* = 34)				Treatment Group (*n* = 33)				*p*-Value
			Baseline	12 Weeks	Diff	*p*-Value	Baseline	12 Weeks	Diff	*p*-Value	
Peak Torque (N·m)	Left	Flexor	33.6 ± 12.6	34.4 ± 12.3	0.83 ± 11.0	0.662	31.0 ± 12.1	34.1 ± 17.5	3.11 ± 18.3	0.336	0.541
		Extensor	72.9 ± 18.6	73.8 ± 18.8	0.84 ± 12.4	0.695	73.6 ± 21.2	72.9 ± 22.8	−0.72 ± 12.8	0.748	0.614
	Right	Flexor	35.8 ± 12.7	33.9 ± 14.2	−1.85 ± 9.3	0.255	30.1 ± 12.9	36.3 ± 18.4	6.18 ± 15.3	0.027	0.013
		Extensor	74.7 ± 16.7	73.5 ± 15.5	−1.24 ± 9.8	0.467	74.7 ± 20.8	75.7 ± 19.6	1.02 ± 13.1	0.657	0.425
Peak TQ/BW (%)	Left	Flexor	57.7 ± 19.5	59.9 ± 20.3	2.26 ± 18.2	0.475	50.7 ± 18.4	55.1 ± 24.4	4.36 ± 28.1	0.379	0.718
		Extensor	125.8 ± 27.0	128.1 ± 29.4	2.25 ± 21.3	0.542	118.8 ± 28.6	118.1 ± 30.3	−0.63 ± 24.6	0.883	0.610
	Right	Flexor	62.1 ± 21.6	58.9 ± 23.4	−3.13 ± 16.7	0.283	49.2 ± 19.8	58.9 ± 27.7	9.71 ± 25.8	0.038	0.019
		Extensor	128.8 ± 22.5	127.4 ± 22.8	−1.44 ± 16.1	0.606	120.1 ± 26.4	123.3 ± 24.3	3.22 ± 18.7	0.330	0.278

Values are presented as the mean ± standard deviation. *p*-Values were determined by an independent *t*-test or chi-square test.

**Table 3:** Effects of tannase-treated green tea extract on grip strength.

			Control Group (*n* = 34)				Treatment Group (*n* = 33)				*p*-Value
			Baseline	12 Weeks	Diff	*p*-Value	Baseline	12 Weeks	Diff	*p*-Value	
Hand grip		Left	27.7 ± 6.3	26.9 ± 5.8	−0.81 ± 2.9	0.112	26.6 ± 6.11	26.2 ± 5.6	−0.44 ± 2.9	0.388	0.602
		Right	29.7 ± 6.1	28.1 ± 5.6	−1.57 ± 3.0	0.004	28.2 ± 4.8	28.1 ± 5.2	−0.15 ± 2.5	0.726	0.037

Values are presented as the mean ± standard deviation. *p*-Values were determined by an independent *t*-test or chi-square test.

**Table 4:** Effects of tannase-treated green tea extract on body muscle mass.

			Control Group (*n* = 34)				Treatment Group (*n* = 33)				Value
			Baseline	12 Weeks	Diff	*p*-Value	Baseline	12 weeks	Diff	*p*-Value	
Body muscle mass (g)	Arm	Left	1863.0 ± 462.2	1783.3 ± 453.6	−79.7 ± 148.0	0.004	1888.6 ± 487.5	1798.1 ± 453.8	−90.4 ± 357.7	0.156	0.874
		Right	1975.0 ± 432.4	1891.9 ± 464.2	−83.0 ± 175.7	0.009	1998.8 ± 441.8	1929.5 ± 453.1	−69.2 ± 378.4	0.301	0.850
	Trunk		18,159.7 ± 2972.4	18,961.9 ± 3381.5	802.3 ± 1228.3	0.001	18,486.8 ± 3766.2	19,845.1 ± 3143.4	1358.3 ± 2801.1	0.009	0.301
	Leg	Left	5304.7 ± 1077.6	5312.3 ± 1111.6	7.6 ± 227.2	0.847	5205.1 ± 1290.1	5406.6 ± 974.0	201.4 ± 916.6	0.216	0.246
		Right	5431.3 ± 1100.5	5396.1 ± 1119.0	−35.2 ± 277.6	0.465	5283.2 ± 1191.9	5421.1 ± 920.2	137.9 ± 899.3	0.385	0.297
	Total		35,660.1 ± 6099.3	36,052.9 ± 6350.5	392.8 ± 1188.2	0.063	35,789.5 ± 7243.6	37,147.5 ± 5707.7	1358.0 ± 5272.2	0.149	0.312

Values are presented as the mean ± standard deviation. *p*-Values were determined by an independent *t*-test or chi-square test.

**Table 5:** Effects of tannase-treated green tea extract on hormones.

	Control Group (*n* = 34)				Treatment Group (*n* = 33)				*p*-Value
	Baseline	12 Weeks	Diff	*p*-Value	Baseline	12 Weeks	Diff	*p*-Value	
Follistatin (pg/mL)	2109.7 ± 539.6	2225.6 ± 739.5	115.9 ± 558.3	0.235	1980.2 ± 720.1	2020.2 ± 633.0	40.1 ± 517.0	0.659	0.566
Myostatin (ng/mL)	1.08 ± 0.61	1.10 ± 0.94	0.02 ± 0.47	0.677	1.23 ± 1.27	1.00 ± 0.66	−0.22 ± 0.62	0.007	0.045

Values are presented as the mean ± standard deviation. *p*-Values were determined by an independent *t*-test or chi-square test.

## Data Availability

Data is contained within the article.

## Supplementary Materials

The following are available online at https://www.mdpi.com/article/10.3390/antiox10071026/s1. Table S1. Description of placebo and treatment products; Table S2. Effects of tannase-treated green tea extract on biochemical parameters of subjects in the control and treatment groups assessed at the baseline and after 12 weeks; Table S3. Effects of tannase-treated green tea extract on safety biomarkers of subjects in the control and treatment groups assessed at the baseline and after 12 weeks.
