# Supplementary material for: (−)-Epicatechin-Enriched Extract from Camellia sinensis Improves Regulation of Muscle Mass and Function: Results from a Randomized Controlled Trial
Source: Antioxidants (Basel). 2021 Jun 25;10(7):1026. doi: 10.3390/antiox10071026 (PMC8300738; doi:10.3390/antiox10071026)
Supplement: Supplementary file 1 [file antioxidants-10-01026-s001.zip › antioxidants-1232569-supplementary.pdf]

Supplementary 1. Description of the placebo and treatment products

		Placebo product	Treatment product
Ingredients and content (%)	Tannase-treated green tea extract	-	83.30
	Cyclodextrin	16.60	16.65
	Magnesium stearate	0.05	0.05
	Microcrystalline cellulose	83.30	-
	Caramel color	0.05	-
Features		Brown capsule	
Packing		One capsule (360 mg) individually packed	

Supplementary 2. Effects of tannase-treated green tea extract on biochemical variables

	Control group (n=34)			Treatment group (n=33)			*p*-value
	Baseline	12 weeks	*p*-value	Baseline	12 weeks	*p*-value	
hs-CRP (mg/L)	1.32 ± 4.64	0.78 ± 1.64	0.486	0.94 ± 1.71	1.63 ± 4.84	0.384	0.266
IL-6 (pg/mL)	2.23 ± 2.19	2.07 ± 1.88	0.378	1.88 ± 1.80	1.48 ± 0.69	0.200	0.490
IL-8 (pg/mL)	18.75 ±10.51	19.44 ± 12.40	0.529	14.88 ± 3.66	15.65 ± 4.99	0.273	0.951
IGF-1 (ng/mL)	148.30 ± 42.12	150.73 ± 45.62	0.715	157.34 ± 41.30	156.94 ± 38.41	0.935	0.728
Cortisol (µg/dL)	0.13 ± 0.06	0.15 ± 0.06	0.152	0.16 ± 0.07	0.17 ± 0.11	0.566	0.823

Values are presented as mean ± standard deviation. *p*-values were measured by an independent t-test

Supplementary 3. Effects of tannase-treated green tea extract on safety parameters

	Control group (n=40)			Treatment group (n=40)			*p*-value[2)]
	Baseline	12 weeks	*p*-value[1)]	Baseline	12 weeks	*p*-value[1)]	
WBC ($\times 10^3/\mu L$)	5.67 ± 1.22	5.35 ± 0.9	0.018	5.89 ± 1.25	5.41 ± 1.15	0.001	0.396
RBC ($\times 100^3/\mu L$)	4.38 ± 0.38	4.39 ± 0.35	0.819	4.58 ± 0.37	4.67 ± 0.36	0.328	0.706
Hemoglobin (g/dL)	13.47 ± 1.01	13.57 ± 1.04	0.372	13.81 ± 1.06	13.90 ± 1.10	0.284	0.943
Hematocrit (%)	39.98 ± 3.07	40.28 ± 2.91	0.407	40.85 ± 2.96	41.28 ± 2.98	0.095	0.752
Platelet ($\times 10^3/\mu L$)	238.25 ± 52.12	245.38 ± 51.14	0.094	243.10 ± 45.71	248.18 ± 46.25	0.208	0.722
ALP (IU/L)	64.73 ± 18.43	66.30 ± 19.70	0.287	75.83 ± 15.67	76.48 ± 18.80	0.643	0.648
Gamma-GT (IU/L)	18.98 ± 16.35	20.40 ± 17.07	0.404	21.33 ± 13.06	21.43 ± 12.52	0.926	0.510
AST (IU/L)	26.53 ± 6.30	27.15 ± 7.66	0.540	26.20 ± 8.02	25.70 ± 8.63	0.763	0.562
ALT (IU/L)	26.58 ± 10.37	26.48 ± 10.32	0.939	26.30 ± 14.40	23.90 ± 8.94	0.322	0.401
Total bilirubin (mg/dL)	0.85 ± 0.29	0.80 ± 0.23	0.187	0.89 ± 0.36	0.87 ± 0.44	0.490	0.694
Total protein (g/dL)	7.29 ± 0.33	7.22 ± 0.30	0.140	7.27 ± 0.38	7.32 ± 0.36	0.348	0.085
Albumin (g/dL)	4.43 ± 0.22	4.41 ± 0.22	0.402	4.45 ± 0.20	4.48 ± 0.18	0.242	0.161
BUN (mg/dL)	14.48 ± 3.43	15.10 ± 3.79	0.298	15.95 ± 4.80	15.65 ± 4.23	0.580	0.246
Creatinine (mg/dL)	0.69 ± 0.12	0.69 ± 0.14	0.536	0.66 ± 0.17	0.69 ± 0.15	0.262	0.201

Glucose (mg/dL)	90.93 ± 8.48	88.85 ± 8.84	0.082	92.73 ± 7.45	90.25 ± 8.70	0.069	0.821
CK (IU/L)	115.10 ± 57.58	109.20 ± 44.98	0.401	101.23 ± 44.22	117.18 ± 52.91	0.315	0.208
LD (IU/L)	420.73 ± 72.99	418.90 ± 77.68	0.835	393.45 ± 69.81	400.80 ± 54.38	0.323	0.423
SG (1.005~1.030)	1.02 ± 0.01	1.02 ± 0.01	0.941	1.02 ± 0.01	1.02 ±0.01	0.967	0.937
pH (4.5~9.0)	6.19 ± 0.78	6.18 ± 0.81	0.936	6.21 ± 0.91	6.13 ± 0.94	0.531	0.719

Values are presented as mean ± standard deviation.

1) *p*-value was measured by a paired t-test

2) *p*-value was measured by an independent t-test
